# Do public nursing home care providers deliver higher quality than private providers? Evidence from Sweden

**DOI:** 10.1186/s12913-017-2403-0

**Published:** 2017-07-14

**Authors:** Ulrika Winblad, Paula Blomqvist, Andreas Karlsson

**Affiliations:** 1 Department of Public Health and Caring Sciences, Uppsala University, Uppsala, Sweden; 2 Department of Government, Uppsala University, Uppsala, Sweden; 3 Department of Public Health and Caring Sciences, Uppsala University, Uppsala, Sweden

**Keywords:** Privatization, Nursing homes, Quality of care, New public management, For-profit care

## Abstract

**Background:**

Swedish nursing home care has undergone a transformation, where the previous virtual public monopoly on providing such services has been replaced by a system of mixed provision. This has led to a rapidly growing share of private actors, the majority of which are large, for-profit firms. In the wake of this development, concerns have been voiced regarding the implications for care quality. In this article, we investigate the relationship between ownership and care quality in nursing homes for the elderly by comparing quality levels between public, for-profit, and non-profit nursing home care providers. We also look at a special category of for-profit providers; private equity companies.

**Methods:**

The source of data is a national survey conducted by the Swedish National Board of Health and Welfare in 2011 at 2710 nursing homes. Data from 14 quality indicators are analyzed, including structure and process measures such as staff levels, staff competence, resident participation, and screening for pressure ulcers, nutrition status, and risk of falling. The main statistical method employed is multiple OLS regression analysis. We differentiate in the analysis between structural and processual quality measures.

**Results:**

The results indicate that public nursing homes have higher quality than privately operated homes with regard to two structural quality measures: staffing levels and individual accommodation. Privately operated nursing homes, on the other hand, tend to score higher on process-based quality indicators such as medication review and screening for falls and malnutrition. No significant differences were found between different ownership categories of privately operated nursing homes.

**Conclusions:**

Ownership does appear to be related to quality outcomes in Swedish nursing home care, but the results are mixed and inconclusive. That staffing levels, which has been regarded as a key quality indicator in previous research, are higher in publicly operated homes than private is consistent with earlier findings. The fact that privately operated homes, including those operated by for-profit companies, had higher processual quality is more unexpected, given previous research. Finally, no significant quality differences were found between private ownership types, i.e. for-profit, non-profit, and private equity companies, which indicates that profit motives are less important for determining quality in Swedish nursing home care than in other countries where similar studies have been carried out.

## Background

One of the most common forms of privatization in the area of social services is *contracting*, which implies that public authorities delegate tasks to private actors in exchange for public funds and that the relationship between the parties is regulated through formal contracts [1, 2]. An often discussed challenge with contracting is how the quality of the services provided by private actors can be monitored by public authorities. While privatization is often believed to enhance efficiency, it has also been argued that private providers are more prone than public to lower quality levels in order to reduce costs and generate profit [3]. This problem of so-called *shirking* has been argued to be particularly salient in areas where quality aspects are hard to formulate accurately in contracts, a condition known as incomplete contracting [4–7]. Nursing home care is a type of service believed to be especially vulnerable to incomplete contracting due to its interpersonal nature, the need for flexibility to meet changing care needs, and the difficulties in specifying quality requirements. Despite this, nursing home care has been one of the welfare sectors where contracting has been practiced most widely in recent decades [2, 8].

Given that incomplete contracting is understood as a condition vulnerable to economic exploitation, it can be expected that contractors with profit motives have stronger incentives to exploit such situations than other actors. Previous studies have shown that nursing homes owned by for-profit companies tend to have lower quality than homes with public and non-profit owners [9–13]. Thus, ownership can be seen as a potential explanatory factor behind quality differences in nursing home care, particularly in settings where private actors compete for public contracts on the basis of price. By the same logic, we could expect nursing homes operated by companies guided by short term profit, such as so-called private equity companies, to be even more prone to exploit incomplete contracts by reducing quality in order to lower costs. Organizations with no profit motive, on the other hand, can be expected to have less incentives to do so. So far, however, the relationship between ownership and care quality in nursing homes has not been fully investigated, as most studies have focused on two ownership categories only, for-profit and non-profit. Differences between public and private providers and various kinds of private ownership such as regular for-profit companies and private equity companies have thereby been neglected. Furthermore, the literature on the relationship between ownership and quality in nursing home care has hitherto treated cases foremost in North America, despite the fact that contracting in this sector has become increasingly common in Europe and other parts of the world [8].

In this paper, we test the proposition that ownership in nursing home care affects quality levels by using data from Sweden. Sweden can be seen as an interesting case in this regard, because virtually all nursing home care is still provided through a publicly regulated and tax-funded system, even though actual care services are carried out by a mixture of public, non-profit, and for-profit providers (some of which are private equity companies). This implies that in Sweden, all care providers, regardless of ownership, operate within the same regulatory framework. Another reason to study effects of ownership type on quality in nursing home care in Sweden is the unique dataset on quality in nursing homes available in this case, particularly for the period 2007–2011. In these years, the Swedish National Board of Health and Welfare (NBHW) collected data from all nursing care units in Sweden, in total about 2700, on a range of quality indicators measuring both processual and structural aspects of care quality. Hence, extensive quality data is available for both publicly and privately operated nursing homes, and the latter category can also be divided on the basis of different private ownership categories, including for-profit, non-profit, and private equity companies. This provides for a unique opportunity to investigate the relationship between ownership type and nursing home care quality.

### Does ownership matter for quality in nursing home care?

It is usually assumed that profit-seeking companies have stronger incentives than public or non-profit organizations to strive for cost reductions that lead to quality deterioration in the provision of public services [5, 14]. The main argument behind this assumption is that public owners have weaker incentives to implement cost reductions than private owners since they will only get a fraction, if anything, of the economic surplus, while private owners will get the whole surplus. Shleifer argues that the incentives to strive for cost reductions are stronger when (1) competition is limited (2) consumer choice is ineffective and (3) reputational mechanisms are weak [14]. The market for nursing home care is characterized by all three of these mechanisms. First, it can be argued that competition is generally weak when public service provision is contracted out. In public procurement processes, bidders are typically invited to compete for contracts, but once the contract is signed, there is essentially no further competition. Strong initial competition for contracts may reduce the providers’ profits by forcing down prices but does not restrain them from making cost reductions on non-contractible quality aspects once the contract is won. Second, for competition to have the desired effect to uphold quality, consumers must be able to make *informed* choices, i.e. use their exit strategy when suppliers are skimping on quality. Research has shown that older people are particularly ineffective consumers when it comes to choosing welfare services. A large percentage of the population over the age of 75 suffers from illness or cognitive impairments making it hard for them to make informed choices [15]. For the mechanism of consumer choice to have a quality-promoting effect within publicly funded welfare service systems, there also needs to be enough excess capacity in the system to make it possible for users to obtain the services they most prefer [16].

Third, a strong reputational mechanism could mitigate the risks of quality cutting in the market for nursing homes. In conventional markets, companies often have incentives to build relationships with consumers and providers by delivering high quality even for dimensions that are hard to contract or to observe, as it strengthens their reputation and sales in the long run. This sort of informal mechanism is, however, hard to rely on in cases of public contracting, at least in the relatively strict form that is employed in nursing home care in Sweden. Swedish public procurement law, drawing on EU directives, prohibits, for instance, that contracting authorities take factors such as previous experiences with a provider into account in the next procurement round, which undermines the mechanism of reputation-building. Thus, in the market for public procurement of nursing homes there seems to be no strong mechanism linking suppliers’ performance today to future demand; both the consumer and the buyer are ineffective in constraining the providers’ incentives to cut costs and thereby decrease quality. On the basis of this reasoning, it can be hypothesized that private nursing home care providers, facing stronger budget constraints and, in many cases, demands for profit, will be more likely to shirk on quality in order to reduce costs. This can be hypothesized to be true also, but to a lesser extent, in the case of private non-profit providers, as these are exposed to the same cost-cutting pressure when they compete for public contracts as for-profit firms even though their owners are less likely to expect a profit. Along the same lines, it can be presumed that the higher the expectation to generate profit on the part of private companies, the more likely they are to compromise quality in order to reduce their costs [11]. Therefore it can be expected that nursing homes owned by so-called private equity companies, that is, owners with a strong demand for short-term profit, will be more likely than the average company to reduce quality. Private equity companies are known for investing in markets with high profit potential, with the objective to improve the financial results over a short time horizon in order to resell the company [17].

Based on the reasoning above, the following hypotheses can be formulated for testing in the empirical analysis:

- **H1.** Public nursing home care providers deliver higher quality than private providers
- **H2.** Non-profit nursing home care providers deliver higher quality than for-profit providers
- **H3.** For-profit nursing home care providers deliver higher quality than private equity providers

### Previous research on ownership and quality in nursing home care

The expectation that ownership may exert influence on quality in nursing home care is supported by previous research in the field. A number of American studies have found that for-profit ownership is related to lower quality than non-profit ownership [9, 10, 12, 18]. In general, non-profit nursing homes have been shown to have higher staffing levels, lower staff turnover and better trained staff compared to for-profit providers. They also have better quality outcomes, such as lower prevalence of pressure ulcers, less use of physical restraints, and fewer deficiencies in governmental regulatory assessments. Furthermore, it has also been shown that for-profit providers tend to deliver lower quality care to residents that have no living relatives or are suffering from dementia, i.e. to persons who have a low "voice" [19]. This implies that profit incentives put weaker consumers at a disadvantage. In a study carried out by Harrington in 2012 it was investigated whether the largest long-term care chains in the U.S. had poorer quality after being purchased by private private equity companies. It was found in this study that there was little change in staffing levels, but that the total number of deficiencies increased after the changes in ownership [11].

Most previous studies examining the relationship between ownership and nursing home care quality have relied on cross-sectional designs, which suggests that their conclusions could be biased due to unobserved factors influencing nursing home quality [18, 20, 21]. In an attempt to isolate the causal effect of ownership change, American researchers Grabowski & Stevenson used panel-data from 1993 to 2004 covering changes in ownership from for-profit to non-profit, and vice versa. Their results, indicating that facilities changing from non-profit to for-profit ownership generally declined in quality while facilities changing ownership from for-profit to non-profit improved, seemed to confirm the relation between ownership and quality observed in previous studies [18].

As Spilsbury et al. points out, research on nursing home quality has hitherto been undertaken primarily in the U.S. [21]. There are, however, a few studies conducted in Sweden which have compared performances between privately and publicly operated nursing homes. The results of these indicate a similar pattern as in the U.S., particularly with regards to the staffing levels and other structural quality indicators, such as staff education and full-time employment [22, 23]. For instance, Stolt et al., using data from 2007, found that the number of employees per resident was significantly lower (−9%) in nursing homes operated by private owners (including both non-profit and for-profit actors) than publicly operated facilities [22]. A similar result was found in the study conducted by the National Board of Health and Social Welfare (NBHW) in 2012 [23]. On the other hand, both Swedish studies also found that private providers scored higher than their public counterparts on quality indicators labeled as processual, such as the proportion of residents participating in the formulation of their care plan; the proportion of residents with a reasonable duration between evening meal and breakfast; and the proportion of residents offered different food alternatives. Worth noting, however, is that none of these studies compared for-profit with non-profit nursing homes. Nor has any scientific study investigated the role of private equity ownership in nursing home care in Sweden despite this being the most form of private ownership in recent decades.

In sum, the previous research on quality in relation to ownership in nursing home care provides fairly consistent evidence that for-profit ownership is associated with lower quality of care than both non-profit and public ownership, at least in an American context.

In particular, nursing homes operated by private for-profit providers appear to have lower staffing levels compared to publicly owned homes, a result found in Swedish studies as well. This tendency is interesting as a large stream of research emphasizes staffing levels as a particularly important determinant of quality of care in nursing homes, particularly when it comes to outcome-related quality indicators such as ulcers and fall injuries [11, 20, 24, 25]. These findings support our general hypothesis that care providers with profit motives are more likely to shirk on quality. Still, we do not know if the same pattern exists in Sweden where, so far, no distinction has been made in the scientific literature between non-profit and for-profit private providers and where the relation between ownership and quality has been shown to be more ambiguous.

### Nursing home care in Sweden: Public funding and mixed provision

Nursing home care in Sweden is provided by 290 local governing units, the so-called municipalities. The services are funded by the municipalities themselves through local income taxation, and are in most cases also provided directly by the municipalities. A legal change in 1992 made it possible for the municipalities to contract out nursing home care provision to private actors including for-profit companies [26]. Since then, the share of residents living in private nursing homes has increased steadily, reaching 20.5% in 2016 [27]. This implies that the private providers are still funded exclusively by the municipalities and subjected to direct regulatory control through contracts in the same way as public providers. In addition, they are bound to comply with the same national public regulation as municipal care providers, with regard to quality requirements, user safety regulations, and audit measures. In contrast to most other welfare systems outside the Nordic region, the Swedish elder care system is organized on the basis of universality. This means that nursing home care services are financed on the principle of solidarity  through income taxation but also that the distribution is organized on the basis of assessed need [26]. Virtually no private market for such services exists in Sweden. In principle, all Swedish citizens (or permanent residents) have access to publicly funded nursing home care services at heavily subsidized rates. User fees have a maximum ceiling and are tied to income. Needs assessment is carried out by social workers at the municipal social service authorities (*Socialtjänsten*) and it is also the municipal social services that decide on the placement of users in individual homes. This means that the private providers operating within the municipal systems of nursing home care provision cannot themselves decide which users to accept, but are obliged to take in all users placed by the municipality. Users placed at privately operated institutions pay the same user fees as in the public homes. In 2015, 16.2% of the population above 65 years of age received some form of elderly care services from the municipalities, while 4.2% lived in a nursing home [28]. As home care services for the elderly are well-developed in Sweden, and this has been the preferred form of care for this group since the 1970s, those assessed to have a need for nursing home care have relatively extensive social and medical needs.

The process through which nursing home care provision is delegated to private actors may be denoted as *competitive contracting*. This implies that interested parties compete for public contracts based on a combination of price and quality. The experience in Sweden so far is that prices have played a determining role when contracts are awarded, even if most municipalities have some form of quality “base line” which all tenders must pass in order to be considered. According to Swedish competition law, contracts between the municipality and private providers cannot be entered into without a transparent and non-discriminatory selection process, which means that non-profit organizations are obliged to compete for municipal contracts on the same basis as for-profit firms. Notably, what is contracted out in the Swedish case is the *operation* of nursing homes, which implies that the facilities are in most cases still owned by the municipalities. Swedish  law also stipulates that the staff employed at the nursing home in question must retain their employment for at least one year after a new provider takes over the operation. Contracts are relatively short, typically 3–4 years, with the possibility of a single extension. In practice, this means that most of the staff at nursing homes where operations are contracted out remain when there is a change in management, for instance from the municipality to a private company. It is important to note, that contracting out nursing homes is voluntary on part of the municipalities and was done in about one third of them in 2016. There is also large regional variation in this regard as some municipalities chose to contract out all local nursing homes, i.e. 100%, whereas others chose to contract out only a limited number or none at all. 

Among private nursing home providers in Sweden, three ownership categories can be identified: non-profit, for-profit, and private equity owned nursing homes, which is a subset of the for-profit category. Non-profit refers to nursing homes run by private organizations without profit motives and with a social orientation, such as churches or charity organizations [29]. The non-profit sector of nursing home care in Sweden is very small, making up only 2.6% of all nursing homes and 10.7% of the privately operated homes in 2015. The majority (89%) of private nursing homes are instead operated by for-profit companies or private equity companies (data from Statistics Sweden from 2015, authors’ own analysis). The ownership category for-profit refers to nursing homes that are operated by for-profit firms, typically stock-holding companies but not owned by private equity companies. Private equity owned refers to nursing homes run by companies that are owned by so-called private equity companies, that are known for investing in markets with high profit potential.

The Swedish market for nursing home care is heavily consolidated. In 2012, four private equity companies controlled 52% of the private market for nursing homes, representing about 11% of the total market [16]. In 2012, the social care sector (care for the elderly and disabled) showed a return on total capital of 15%, which was quite high compared to other sectors in the same year. The median return on equity in the same sector was 38.5%, a high figure compared to the corresponding figure of 19% for the service sector as a whole (data from Statistics Sweden for 2013). Thus, the Swedish nursing home care sector is clearly attractive to private investors, even if the majority of care providers (nearly 80%) are still public.

## Methods

### Aim and setting of the study

The aim of this study is to investigate the relationship between ownership type and care quality in nursing home care in the case of Sweden. From a methodological perspective, Sweden can be seen as a least likely case. It may be expected that differences in quality between different types of care providers are relatively small, given the high level of public regulation, the public funding structure, and the high social and political premium placed on equitable care standards among all types of providers. The least likely case selection implies that if quality differences are found between various ownership categories in the Swedish case, they are likely to exist in other countries as well [30].

### Data and material

The data used in the study originate from the Swedish National Board of Health and Welfare (NBHW), an expert government agency in the welfare area, which conducts surveys covering a range of quality indicators of all nursing homes in Sweden on a yearly basis. The collection of data is organized so that every nursing home regardless of ownership, receives a questionnaire asking management to provide statistics on the listed quality indicators.

Following Donabedian’s well-known distinction, we differentiate in the analysis between structural and processual quality indicators [31]. Structural quality refers to institutional characteristics such as staffing and facilities, measured, for instance, as the number of employees per resident, prevalence of full-time employment or access to a kitchen. Processual quality indicators measure the activities performed by the staff, such as frequency of medication reviews or risk assessments for pressure ulcers, or malnutrition. By distinguishing between structural and processual quality measures we can obtain a more precise understanding of in what ways different types of providers make trade-offs between cost and quality.^1^

The data used in the study was collected by NBHW in October 2010 and 2011. The reason for why we choose data from these years is that it was the only period when the quality indicator Employees per resident was used, a quality measure that has often been used in previous studies. The data from 2011 comprised 2324 publicly operated and 386 privately operated nursing homes, out of which 201 were private equity homes, 123 private for-profit homes and 62 private non-profit homes. In the analysis we used seven structural measures and seven processual measures, see Table 1. All of these, except for four structural measures, originate from the 2011 survey. These four measures, Employees per resident, Hourly employment, Full time employment and Advanced competence, were collected from the 2010 survey, since these indicators were not measured in 2011 by the NBHW.

**Table 1:** Description of the quality indicators

Quality indicator	Description	Quality dimension
Employees per resident^a^	Number of employees measured as full work years divided by total number of residents	Structural
Hourly employment^a^	Percentage of full work years performed by part-time employees paid per hour	Structural
Full-time employment^a^	Percentage of employees working at least 85% of full time	Structural
Employee turnover	Percentage of employees who had quit during the preceding year	Structural
Advanced competence^a^	Percentage of employees with a college or university health care education	Structural
Basic competence	Percentage of employees with an upper secondary school health care education	Structural
Individual accommodation and kitchen	Percentage of residents with individual (or shared with spouse, partner, or relative) accommodation including cooking facilities	Structural
Participation	Percentage of residents or appointed representatives participating in the formulation of the care plan	Processual
Updated care plan	Percentage of residents who had an updated care plan (not older than six months)	Processual
Nightly fast	Percentage of residents with a nightly fast of 11 h or less	Processual
Medication review	Percentage of residents that have had his/her prescribed medication assessed within the last 12 months	Processual
Screening for falls	Percentage of residents assessed for risk of falling	Processual
Screening for pressure ulcers	Percentage of residents assessed for risk of pressure ulcers	Processual
Screening for malnutrition	Percentage of residents assessed for risk of malnutrition	Processual

^a^For the indicators *Employees per resident*, *Hourly employment*, *Full-time Employment*, and *Advanced competence*, data from 2010 are used since these indicators were not measured in 2011 by the NBHW

As the purpose of this paper is to investigate whether ownership affects quality of care, we subdivided ownership into four categories with regard to mode of provision: (1) Public; (2) For-profit; (3) Non-Profit; and (4) Private Equity. As NBWH’s data does not divide private homes into all these categories, but only provides a dichotomous classification of facilities into public and private ownership, we used company websites and business registry databases to determine type of ownership. In order to test hypothesis 1, that publicly operated nursing homes have higher quality than private ones, we also kept the category “private” in the NBHW data which contains all three types of private actors: for-profit, non-profit and private equity. Thus, five ownership categories are presented in the tables.

### Analytical approach

We performed OLS regressions to compare mean quality differences between the different ownership groups. Dummy variables were constructed for each ownership group, using public nursing homes as a reference group (Table 3). The quality indicators were treated as dependent variables and the ownership categories as independent variables. To test hypothesis 2 and 3, for-profit homes and private equity homes were used as reference groups respectively.

Multiple OLS regressions were run to control for confounding factors that could obscure the relationship between quality of care and ownership. For example, it has been shown that privatization is more prevalent in larger cities and suburbs [32]. Privatization also tends to co-vary with political ideology as municipalities governed by right-wing parties tend to contract out nursing homes to a larger extent. In municipalities with left-wing parties, on the other hand, a strained financial situation seems to co-vary with increased privatization [33]. For these reasons all OLS models were controlled for municipality population density, political majorities, and socio-demographic, and financial differences (Appendix 1). We have adjusted the analysis for multiple testing.

## Results

To start with, we compared the mean quality for all quality indicators across all five ownership types, see Table 2. As can be seen, publicly operated nursing homes had more employees per resident, fewer staff with hourly employment, and a higher share of homes offering individual accommodation/kitchen. Among the private providers, private equity homes had the lowest values for the indicator Employees per resident, and for-profit homes had the lowest proportion of Employees with hourly employment. Non-profit homes had staffing levels most similar to publicly operated nursing homes. Table 2 also shows that all categories of privately operated nursing homes outperform the publicly operated homes on the processual quality indicators such as Medication reviews or Screening for falls.

**Table 2:** Descriptive statistics of Quality means presented by type of ownership, 2011

Type of quality		Public	Private	Private equity	Private for-profit	Private non-profit
	No. nursing homes	2324	386	201	123	62
Structure	Employees per resident^a^	0.9	0.8	0.8	0.8	0.9
	Hourly employment^a^	13.4	17.3	18.8	14.9	16.6
	Full-time employment^a^	45.5	45.7	46.0	44.4	46.9
	Employee turnover	7.5	8.6	8.1	8.6	10.1
	Advanced competence^a^	9.1	10.5	10.8	10.1	10.3
	Basic competence	84.5	82.4	81.1	83.3	85.0
	Individual accommodation/kitchen	50.9	44.8	46.3	43.9	41.9
Process	Updated care plan	86.7	94.2	93.2	94.8	96.2
	Participation	80.5	90.4	89.6	89.9	93.9
	Nightly fast	89.9	97.1	96.0	98.2	98.3
	Medication review	66.3	82.6	80.9	85.1	82.7
	Screening for falls	62.0	83.5	80.0	86.4	88.8
	Screening for pressure ulcers	57.9	76.3	73.0	77.6	83.9
	Screening for malnutrition	62.1	78.9	75.5	84.1	79.6

Note: ^a^For the indicators Employees per resident, Hourly employment, Full-time Employment, and Advanced competence, data from 2010 are used since these indicators were not measured in 2011 by the NBHW. The data from 2010 comprised 1583 publicly operated nursing homes and 265 privately operated nursing homes (152 private equity, 69 private for-profit, 44 private non-profit)

Table 3 shows the results for the multiple regression analysis of structural and processual quality indicators while controlling for municipality characteristics, thus testing hypothesis 1 that public nursing home care providers perform better than private providers. The analysis shows that privately operated nursing homes are associated with significantly lower staffing levels (−0.07) than public homes. The difference is most pronounced in the case of private equity operated homes, where it is −0.09. This can be seen as a relatively large difference, given that public nursing homes have 0.9 staff per resident. Another structural quality indicator where public homes score higher is individual accommodation/kitchen (−9.15). For the other structural quality indicators there are no statistically significant differences between private and public providers.

**Table 3:** Comparison of quality outcomes between all ownership types, unadjusted and adjusted coefficients, 2011 (reference group = public providers

		All private^a^		Private equity		For-profit		Non-profit	
	N=	386		201		123		62	
Type of quality indicator		unadjusted	adjusted	unadjusted	adjusted	unadjusted	adjusted	unadjusted	adjusted
Structure	Employees per resident^b^	-0,07***	−0.07***	−0,1***	−0.09***	−0.04	−0.04	−0,03	−0.03
		(0.01)	(0.01)	(0.01)	(0.02)	(0.02)	(0.02)	(0.02)	(0.03)
	Hourly employment^b^	3,89***	1.84	5,42***	3.28	1,48	−0.48	3,2	0.73
		(0.81)	(0.84)	(1.14)	(1.15)	(1.42)	(1.44)	(1.06)	(1.21)
	Full-time employment^b^	−0.21	−2.63	0.57	−2.56	−1.09	−2.1	1,42	−4.5
		(1.26)	(1.22)	(1.63)	(1.55)	(2.21)	(2.03)	(3.11)	(2.77)
	Employee turnover	1.07	0.92	0.57	0.3	1,05	0.86	2,58	1.47
		(0.93)	(1.11)	(1.15)	(1.30)	(1.69)	(1.91)	(2.46)	(2.60)
	Advanced competence^b^	1,46**	−0.01	1,74**	0.14	1.02	−0.16	1,27	−0.69
		(0.38)	(0.42)	(0.46)	(0.55)	(0.78)	(0.75)	(0.75)	(0.81)
	Basic competence	−2.06	−1.36	−3.41	−2.68	−1.15	−0.52	0,52	1.31
		(0.83)	(0.88)	(1.10)	(1.13)	(1.35)	(1.40)	(2.14)	(2.24)
	Individual accommodation/kitchen	−6.08	−9.15**	−4.63	−6.43	−7.00	−11.3	−8,97	−13.4
		(2.74)	(2.95)	(3.67)	(3.85)	(4.60)	(4.62)	(6.35)	(6.50)
Process	Updated care plan	7,53***	6.11***	6,56***	4.89***	8,09***	6.68***	9,54***	7.71***
		(0.88)	(0.93)	(1.21)	(1.23)	(1.36)	(1.49)	(1.32)	(1.78)
	Participation	9,90***	7.72***	9,11***	6.67***	9,38***	7.17*	13,4***	11.4***
		(1.17)	(1.26)	(1.56)	(1.58)	(1.95)	(2.13)	(1.89)	(2.32)
	Nightly fast	7,21***	2.85	6,09**	1.45	8,32***	3.75	8,41***	2.25
		(1.12)	(1.26)	(1.63)	(1.74)	(1.42)	(1.55)	(1.81)	(2.07)
	Medication review	16,2***	15.3***	14,6***	13.5***	18,7***	18.1***	16,4**	15.3*
		(1.79)	(1.98)	(2.43)	(2.59)	(2.70)	(2.85)	(4.07)	(4.44)
	Screening for falls	21,6***	20.2***	18,1***	17.6***	24,4***	23.9***	26,8***	25.9***
		(1.62)	(1.85)	(2.28)	(2.47)	(2.38)	(2.56)	(2.95)	(3.56)
	Screening for pressure ulcers	18,7***	16.1***	15,4***	14.0***	20,0***	19.0***	26,3***	25.0***
		(1.98)	(2.26)	(2.68)	(2.90)	(3.26)	(3.43)	(3.95)	(4.61)
	Screening for malnutrition	16,8***	15.7***	13,4***	12.9***	22,0***	22.3***	17,5**	17.1*
		(1.87)	(2.14)	(2.56)	(2.79)	(2.74)	(2.94)	(4.31)	(4.91)

*p < 0.05 **p < 0.01 ***p < 0.001. *P*-values have been adjusted for multiple testing, i.e. divided by 42 (14 quality indicators*3 ownership groups). Standard errors are in parentheses

^a^‘All private’ includes ‘Private equity’, ‘For-profit’ and ‘Non-profit’

^b^For the indicators Employees per resident, Hourly employment, Full-time Employment, and Advanced competence, data from 2010 are used since these indicators were not measured in 2011 by the NBHW

The data from 2010 comprised of 1583 publicly operated nursing homes and 265 privately operated nursing homes (152 private equity, 69 private for-profit, 44 private non-profit)

Looking at processual quality indicators in the same table, it can be noted that privately operated nursing homes across all ownership types are associated with higher values on processual quality than are public homes. For example, privately operated homes have significant larger percentages of residents that have been screened for falls (20.2 percentage points), pressure ulcers (16.1 percentage points), and malnutrition (15.7 percentage points) than publicly operated nursing homes, when controlling for different municipal characteristics.

To summarize, it appears that privately operated nursing homes have lower staffing levels and fewer individual accommodations than publicly operated homes, indicating lower quality in this particular aspect, but that there are no real differences between the two categories in regard to other structural quality measures. The fact that privately operated homes have a lower level of share of individual (e.g. single) rooms cannot solely be attributed to ownership of the organizations that operate the facilities, as these are in most cases still publicly owned. Publicly operated nursing homes displayed lower processual quality levels than their privately operated counterparts. Taken together, this suggests only weak support for hypothesis 1 and indicates that it is the type of quality (i.e. structural or processual), rather than overall quality levels that distinguishes the public sector from the private.

When it comes to the second hypothesis, which stated that non-profit nursing homes can be expected to deliver higher quality than for-profit providers, and the third hypothesis, which stated that for-profit nursing home care providers can be expected to deliver higher quality than private equity providers, the results were more straight-forward. Contrary to the second hypothesis, the results in Table 4 indicate that there are no statistically significant differences between non-profit and for-profit nursing homes, with regard to either structural or processual quality. Likewise, the results in Table 5 show that there are no statistically significant differences in quality levels of a structural or processual nature between nursing homes operated by for-profit firms and nursing homes operated by private equity firms. Hence, no evidence was found to support hypotheses 2 and 3.

**Table 4:** Comparison of quality outcomes between non-profit and for-profit homes (reference group = for-profit + private equity, adjusted coefficients)

Type of quality indicator		*N* = 62
		adjusted
Structure	Employees per resident^a^	0.04
		(0.03)
	Hourly employment^a^	−1.73
		(1.48)
	Full-time employment^a^	−2.26
		(2.99)
	Employee turnover	0.53
		(2.63)
	Advanced competence^a^	−0.53
		(0.81)
	Basic competence	3.61
		(2.29)
	Individual accommodation/kitchen	−4.15
		(6.79)
Process	Updated care plan	2.74
		(1.69)
	Participation	4.90
		(2.28)
	Nightly fast	1.38
		(2.06)
	Medication review	−4.20
		(4.42)
	Screening for falls	2.90
		(3.28)
	Screening for pressure ulcers	5.27
		(4.45)
	Screening for malnutrition	−2.62
		(4.71)

*p < 0.05 **p < 0.01 ***p < 0.001 Note: Tables 4 and 5: *P*-values have been adjusted for multiple testing, i.e. divided by 14 (14 quality indicators*1 ownership group). Standard errors are in parentheses

^a^For the indicators Employees per resident, Hourly employment, Full-time Employment, and Advanced competence, data from 2010 are used since these indicators were not measured in 2011 by the NBHW

The data from 2010 comprised of 1583 publicly operated nursing homes and 265 privately operated nursing homes (152 private equity, 69 private for-profit, 44 private non-profit)

**Table 5:** Comparison of quality outcomes between for-profit and private equity homes (reference group = private equity, adjusted coefficients)

Type of quality indicator		*N* = 123
		adjusted
Structure	Employees per resident^a^	0.04
		(0.03)
	Hourly employment^a^	−3.11
		(1.82)
	Full-time employment^a^	−0.20
		(2.34)
	Employee turnover	0.46
		(1.96)
	Advanced competence^a^	−0.90
		(0.92)
	Basic competence	2.26
		(1.66)
	Individual accommodation/kitchen	−2.43
		(5.60)
Process	Updated care plan	1.66
		(1.73)
	Participation	0.65
		(2.39)
	Nightly fast	3.07
		(1.96)
	Medication review	3.95
		(3.37)
	Screening for falls	5.91
		(2.95)
	Screening for pressure ulcers	3.95
		(3.93)
	Screening for malnutrition	8.12
		(3.48)

*p < 0.05 **p < 0.01 ***p < 0.001 Note: Tables 4 and 5: *P*-values have been adjusted for multiple testing, i.e. divided by 14 (14 quality indicators*1 ownership group). Standard errors are in parentheses

^a^For the indicators Employees per resident, Hourly employment, Full-time Employment, and Advanced competence, data from 2010 are used since these indicators were not measured in 2011 by the NBHW

The data from 2010 comprised of 1583 publicly operated nursing homes and 265 privately operated nursing homes (152 private equity, 69 private for-profit, 44 private non-profit)

## Discussion

The results of the empirical analysis indicate that ownership does affect nursing home care quality in Sweden, but that this relationship appears to vary across different quality measures. For structural quality measures, like staffing and facilities, differences were found in two cases: *Employees per resident* and *Individual accommodation/kitchen*, where publicly operated homes outperformed all categories of private operated homes. The analysis also showed, however, that privately operated care providers outperformed public ones on several processual quality measures, such as *User Participation*; *Updated care plan*; and *Medication review*. Hence, a relationship between ownership and care quality was found in the case of processual quality but in the opposite direction of the structural quality indicators. This ambiguous finding, could be explained in part by the fact that, as private providers are exposed to market competition, they have strong incentives *both* to reduce costs and demonstrate that they provide high quality. Therefore, it is likely that they strive foremost for quality measures that are less costly to implement, like screening practices or routinely offering users a say when their care plan is established (even if many users may not be in a condition to offer much input, given their health status). Another possible explanation for the higher processual quality of privately operated nursing homes in Sweden is that there has been an economic incentive to introduce screening routines for risks such as fall injuries and pressure ulcers in Sweden, as some local governments have offered financial bonuses for providers who have done so. Given their stronger incentives to reduce costs, private owners can be expected to be more sensitive to such incentives. It should be noted, finally, that some researchers have proposed that structural quality measures, particularly staffing density, are more important for quality outcomes of nursing home care (measured for instance through mortality; pressure ulcurs; or violations of national regulatory standards) than processual quality measures [11, 12, 24–26].

When it comes to the expected quality differences between different ownership types *within* the private sector, no clear evidence of this was found. Non-profit operated nursing homes scored higher than for-profit homes on most quality indicators, but the differences were not statistically significant. Likewise, there were no statistically significant differences in quality levels between regular for-profit homes and private equity homes. This indicates that profit motive is not as important a predictor for care quality in Sweden as it appears to have been in some other cases, such as the U.S. [11, 19]. Taken together, the findings in the paper indicate that ownership may play a role for nursing home care quality in Sweden, but that the *way* in which it matters depends on the definition of quality. Furthermore, it is clear that in Sweden, the main effect with regard to how ownership affects care quality is related to the distinction between public and privately operated nursing homes, rather than between different ownership types within the private sector.

These results are only in part consistent with previous research on the relationship between ownership and care quality. As we have seen, a general finding in this research is that for-profit nursing home care providers have lower staffing levels than non-profit providers [9, 11, 18]. As noted above, this pattern is visible in the Swedish case but was not found to be statistically significant. One possible explanation for this is that the non-profit group was very small in the data set, indicating that observations might have been too few to provide for a sound statistical analysis. Another explanatory factor could be the competitive pressures under which non-profit organizations operate in nursing home care in Sweden, as they are exposed to market competition on the same terms as for-profit companies. In this regard, they are more distinct from publicly operated nursing homes, which in most cases do not face the same price competition and same budget restraints.

Taken together, the findings in the article indicate that ownership is related to quality differences but that the relationship between ownership type and care quality appears weaker and more ambiguous in the Swedish case than in other cases where similar studies have been carried out.

One explanation for this may be that the nursing home sector in Sweden is highly regulated and that privately operated nursing homes are required to follow the same regulations and quality standards as public ones. Social equity in access has been a central goal in Swedish nursing home care, which is manifested in its universal nature, with virtually all nursing home care funded by public authorities. The goal to preserve equity within the system has also led to extensive public regulation in order to ensure that service quality is similar in all types of nursing homes, regardless of ownership. Furthermore, the fashion in which contracting is organized in Sweden implies, as noted above, that private companies manage facilities that are publicly owned and that the staff, in most cases, remain when managerial responsibility shifts from public to private organizations or between private organizations. In this sense, public and private providers in Sweden, operate under relatively homogenous conditions, a fact that may serve to prevent quality shirking on part of private providers.

Another difference between the Swedish case and cases previously studied, may be that the regulatory framework in which contracting takes place in Swedish nursing home care also implies that private companies *are not able to select their users*. As we have seen, nursing home care is allocated in Sweden on the basis of need, which is assessed by municipal social workers. It is also the public social workers who decide *where* an elderly person is to receive care, allocating users at various homes managed by either public or private providers. This means that privately operated nursing homes in Sweden will have the same case mix as public homes, a condition that is rarely found in countries like the U.S. and Canada, where case mixes may vary substantially between the public and private sectors.

A limitation of the study is that, given that the data used are cross-sectional, we cannot statistically establish causality between ownership and care quality, but only examine correlation. In order to perform a causal analysis, one would need panel data on longer periods of time which included ownership changes of nursing homes. A second limitation, as noted above, is that the sample of non-profit nursing homes is quite small, which might obscure differences in quality between this group and the for-profit groups. The most significant strength of the study, however, is that the data is comprehensive; covering all nursing homes in Sweden, and that it is possible to separate between different types of private ownership.

A final question concerns the ability to generalize from the case. Here, we believe that there is reason to be cautious, given the rather distinct features of the Swedish nursing home care model, with its universalistic funding scheme, extensive public regulations, and publicly controlled allocation of users to different homes. These factors indicate that quality differences between different types of providers may be smaller in Sweden than in other cases, where regulatory and financial conditions between the public and private sectors differ more. On the other hand, the finding that there was lower staffing density among non-profit care providers also in the Swedish case suggests that this quantitative difference may be a common feature in nursing home care markets, despite varying conditions in funding and regulation.

## Conclusions

In this paper we asked the question whether quality in nursing home care in Sweden is related to ownership. Sweden was identified as a least likely case in this regard, given that private nursing homes are publicly financed, tightly regulated, and lack the possibility of selecting users. Our results indicate that ownership does seem to be related to quality in nursing home care in Sweden, but that it is an ambiguous relationship, as publicly managed nursing homes had better quality scores with regard to structural quality factors such as staffing and individual accommodation/kitchen, but performed worse than privately managed homes when it came to other types of quality measures, related to processual quality.

Another finding was that profit motive did not appear to play as important a role for determining quality in the Swedish case as it has been shown to do in other countries. Our findings showed no statistically significant differences in quality indicators between private providers with different types of ownership, such as non-profit, for-profit and private equity companies. This means that it may matter less for the quality of the services whether nursing homes are operated by non-profit or for-profit actors in Sweden. This finding appears to contradict previous research but may be related to country-specific factors in the Swedish case with regard to the financing and regulation of the nursing home sector.

## Appendix 1

**Table 6:** Control variables used in the Multiple OLS regressions

Variable	Description	Source
Population density	Municipal population density measured as inhabitants per square kilometer	Statistics Sweden 2009–2011
Right-wing representation	Proportion of right-wing members in the municipal parliament	Statistics Sweden 2006 and 2010
Income	Average municipal income level	Statistics Sweden 2009–2011
Result per capita	Three years average municipal net income or net loss excluding extraordinary incomes or costs divided by the municipal population	Statistics Sweden 2007–2011
Spending	Municipalities’ spending (2011–2010 measured per capita,in 2009 measured per elderly resident) on special housing accommodations	Statistics Sweden 2009–2011

The following parties are defined as right-wing

Moderata Samlingspartiet, Liberalerna, Kristdemokraterna and Centerpartiet

## Footnotes

1 The third type of quality discussed by Donabedian, outcome quality, could not be assessed in the analysis since there were no quality indicators available for outcome quality.

## References

[1] Savas ES (2000). Privatization and public-private partnerships.

[2] Gilbert N (2002). Transformation of the welfare state: the silent surrender of public responsibility.

[3] Prizzia R (2001). Privatization and social responsibility: a critical evaluation of economic performance. Int J Public Sect Manag.

[4] Domberger S, Jensen P (1997). Contracting out by the public sector: theory, evidence, prospects. Oxford Rev Econ Policy.

[5] Hart O, Shleifer A, Vishny RW (1997). The proper scope of government: theory and an application to prisons. Q J Econ.

[6] Hart O, Moore J (1999). Foundations of incomplete contracts. Rev Econ Stud.

[7] Segal I (1999). Complexity and renegotiation: a Foundation for Incomplete Contracts. Rev Econ Stud.

[8] Ranci C, Pavolini E (2013). Reforms in long-term care policies in Europe : investigating institutional change and social impacts.

[9] Hillmer MP, Wodchis WP, Gill SS, Anderson GM, Rochon PA (2005). Nursing home profit status and quality of care: is there any evidence of an association?. Med Care Res Rev.

[10] Comondore VR, Devereaux PJ, Zhou Q, Stone SB, Busse JW, Ravindran NC (2009). Quality of care in for-profit and not-for-profit nursing homes: systematic review and meta-analysis. BMJ.

[11] Harrington C, Olney B, Carrillo H, Kang T (2012). Nurse staffing and deficiencies in the largest for-profit nursing home chains and chains owned by private equity companies. Health Serv Res.

[12] Harrington C, Woolhandler S, Mullan J, Carrillo H, Himmelstein DU (2001). Does investor ownership of nursing homes compromise the quality of care?. Am J Public Health.

[13] Amirkhanyan A (2008). Privatizing public nursing homes: examining the effects on quality and access. Public Adm Rev.

[14] Shleifer A (1998). State versus private ownership. J Econ Perspect.

[15] Meinow B, Parker MG, Thorslund M (2011). Consumers of eldercare in Sweden: the semblance of choice. Soc Sci Med.

[16] Bergman M, Jordahl H (2014). Goda år på ålderns höst ?.

[17] Kaplan SN, Strömberg P (2009). Private equity: leveraged buyouts and private equity. J Econ Perspect.

[18] Grabowski DC, Stevenson DG (2008). Ownership conversions and nursing home performance. Health Serv Res.

[19] Chou SY (2002). Asymmetric information, ownership and quality of care: an empirical analysis of nursing homes. J Health Econ.

[20] Backhaus R, Verbeek H, van Rossum E, Capezuti E, Hamers JPH (2014). Nurse staffing impact on quality of care in nursing homes: a systematic review of longitudinal studies. J Am Med Dir Assoc.

[21] Spilsbury K, Hewitt C, Stirk L, Bowman C (2011). The relationship between nurse staffing and quality of care in nursing homes: a systematic review. Int J Nurs Stud.

[22] Stolt R, Blomqvist P, Winblad U (2011). Privatization of social services: quality differences in Swedish elderly care. Soc Sci Med.

[23] The National Board of Health and Welfare (NBHW). Kommunal eller enskild regi, spelar det någon roll ? – en jämförelse av utförare av vård och omsorg om äldre [Public or private ownership, does it matter? - a comparison of providers within eldercare]. Stockholm: 2012.

[24] Castle NG, Engberg J (2008). Further examination of the influence of caregiver staffing levels on nursing home quality. Gerontologist.

[25] Kim H, Kovner C, Harrington C, Greene W, Mezey M (2009). A panel data analysis of the relationships of nursing home staffing levels and standards to regulatory deficiencies. J Gerontol Ser B Psychol Sci Soc Sci.

[26] Erlandsson S, Storm P, Stranz A, Szebehely M, Trydegård G-B, Meagher G, Szebehely M (2013). Marketising trends in Swedish eldercare: competition, choice and calls for stricter regulation. Mark. Nord. Eldercare a res. Rep. Legis. Overs. Extent consequences.

[27] The National Board of Health and Welfare (NBHW). Statistik om äldre och personer med funktionsnedsättning efter regiform 2016 [Statistics about elderly and people with diabilities by ownership type 2016] 2017.

[28] The National Board of Health and Welfare (NBHW). Statistik om äldre och personer med funktionsnedsättning efter regiform 2016a 2017;1:1–2.

[29] Salamon LM, Anheier HK (1998). Social origins of civil society: explaining the nonprofit sector cross-nationally. Volunt Int J Volunt Nonprofit Organ.

[30] George AL, Bennett A (2005). Case studies and theory development in the social sciences.

[31] Donabedian A (2005). Evaluating the quality of medical care. Milbank Q.

[32] Stolt R, Winblad U (2009). Mechanisms behind privatization: a case study of private growth in Swedish elderly care. Soc Sci Med.

[33] Suzuki K. Marketization of Eldery Care in Sweden. EIJS Work Pap Ser Nr 137 2001.

